# Consumption Cure

**Published:** 1882-01

**Authors:** 


### CONSUMPTION CURE.

**There** will soon be so many methods of curing this formidable malady, it would seem good policy for people to do all they possibly can to become consumptive, on the general principle, that as two distinct diseases do not prevail in the system at one time, and consumption being cured without any difficulty, such as by smelling sugar, smoking arsenic, or less laboriously still, drinking brandy, that being to a large number no trouble at all, not the slightest, in fact rather pleasurable if any thing, the most direct road to health for those who have any thing the matter with them is to get consumption, get cured of it by selecting the most agreeable method of the many proposed, and be permanently well.

A statement was recently made in a European Journal, that arsenic smoking has been successful in the hands of some, in restoring consumptive persons to fulness of flesh and to the soundness of health; this article, in consequence of the reckless and careless manner with which newspapers are "scissored" up, daily becoming more common, has been very extensively copied, and without a warning may do injury. It is a fact that smoking arsenic, or eating it, will give plumpness to the flesh, and so will porter or cod liver oil; it will paint upon the cheek the ruddy hue of health, and so will good brandy; it will remove shortness of breath with astonishing promptitude, and so will a plug of tobacco or opium, or a good breath of ether or laughing gas. We have no objection at all to the arsenic treatment, except that as soon as the patient ceases to use it, he dies; and if he continues its use, he dies any how, only more rapidily than he would with either opium, cod liver oil, porter or brandy.

Let it not be forgotten, that no agent is curative of consumption which does not impart vigor to the digestion, and capacity to the lungs to use a larger amount of that which great nature has expressly provided for their nourishment, the pure air of heaven; and which, as every educated man must know, is the only thing in the wide universe which can put the finishing stroke to that which is then called "*blood*," the only thing which can perfectly unload the blood, when diseased or impure, of that which has rendered it so.

We conclude, therefore, that the best and safest remedies for consumption are pure air, sunshine, exercise, comfortable clothing, and wholesome food. As a rule, medicines should be avoided.

The "Spirometer" is rapidly growing into favor with our best physicians, particularly as a means of diagnosis of lung diseases. There is no doubt but its judicious use is decidedly beneficial, not only to consumptives, but to all persons, and particularly to those who have not opportunity for frequent exercise in the open air, since it brings into action that large portion of the lungs which the air does reach in the act of natural breathing. It in fact supplies food to the lungs.

